# A complicated case of open wound managed by platelet rich plasma

**DOI:** 10.11604/pamj.2021.39.238.29867

**Published:** 2021-08-12

**Authors:** Neha Vinay Chitale, Pratik Arun Phansopkar

**Affiliations:** 1 Department of Musculoskeletal Physiotherapy, Ravi Nair Physiotherapy College, Datta Meghe Institute of Medical Sciences, Sawangi (Meghe), Wardha, Maharashtra, India

**Keywords:** Open wound, platelet rich plasma, road traffic accident, COVID-19

## Image in medicine

A 23-year-old female known case of renal agenesis met with a road traffic accident, patient was unconscious and had an injury on lateral aspect of her ankle. The wound was 7cm in length, 5cm in width and tendons of peroneus longus and peroneus brevis, sural nerve and short saphenous vein were exposed (A). Considering the wound, grafting was the choice of treatment. As the patient was unconscious, investigations were done and subdural bleed of 4mm thickness was found. Wound culture showed infection by Klebsiella. All these factors were responsible for delay in surgery. After the subdural bleed and infection was resolved, grafting was planned but the patient tested positive for COVID-19, and thus was quarantine for 14 days, Hence the surgery delayed. By the time patient tested negative for COVID-19 a total of 28 days were passed; however, the wound was healing rapidly (B). Debridement was done on 29^th^ day (C). Considering the good healing rate, platelet rich plasma (PRP) seemed to be a better option than surgery. Eight (8) PRP sessions were given total to the patient for next 4 weeks with 2 sessions per week. Physiotherapy was given along with PRP 3 sessions per week each session lasting for 45 mins. No significant improvement was observed after first two sessions of PRP (D) but healing rate accelerated post 3^rd^ session (E). Post 8^th^ session the size of wound was significantly reduced and appearance of new normal skin structure was seen (F) and the wound recovered completely.

**Figure 1:**
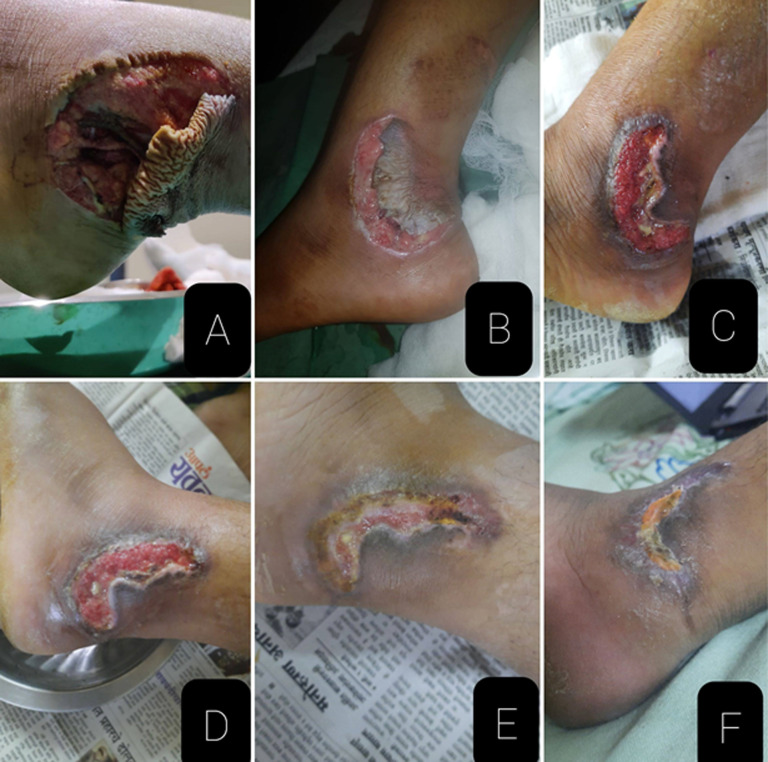
A) open wound at later aspect of ankle; B) 28^th^ day post injury; C) post-debridement on 29^th^ day; D) 1^st^ platelet rich plasma session; E) 4^th^ session of platelet rich plasma; F) 8^th^ session of platelet rich plasma

